# The effect of Kinesio taping on cervical proprioception in athletes with mechanical neck pain—a placebo-controlled trial

**DOI:** 10.1186/s12891-020-03681-9

**Published:** 2020-10-03

**Authors:** Khalid A. Alahmari, Ravi Shankar Reddy, Jaya Shanker Tedla, Paul Silvian Samuel, Venkata Nagaraj Kakaraparthi, Kanagaraj Rengaramanujam, Irshad Ahmed

**Affiliations:** Department of Medical Rehabilitation Sciences, College of Applied Medical Sciences, King Khalid University, Abha, Saudi Arabia

**Keywords:** Neck pain, Kinesio tape, Position sense, Athletes

## Abstract

**Background:**

Neck proprioception is critical in maintaining neuromuscular control in and around cervical joints. Kinesio™ tape may assist in rehabilitating joint position sense. The current study compares Kinesio™ tape’s effects versus a placebo on proprioception in college athletes experiencing mechanical neck pain.

**Methods:**

This study randomized sixty-six athletes with mechanical neck pain into a Kinesio™ tape group (*n* = 33, mean age = 22.73 years) or placebo group (n = 33, mean age = 23.15 years). The Kinesio™ tape group received standard Kinesio™ taping applications with appropriate tension, while the placebo group received taping applications without tension. Outcome measures: The study assessed cervical joint position errors with a cervical range-of-motion (CROM) device, pain intensity with a visual analog scale (VAS), and neck functional disability with a neck disability index (NDI). It tested joint position errors through cervical flexion, extension, rotation left, and rotation right. All the outcome measures were recorded at the baseline and twice more following 3 and 7 days of tape applications.

**Results:**

Multivariate analysis of variance test demonstrated a significant reduction in joint position errors in flexion, extension and right rotation following 3 days and 7 days of tape application among the Kinesio™ tape group. There was a significant main effect of time (*P* < 0.05) for joint position errors in left rotation and VAS after 3 days (*p* > 0.05), NDI after 3 and 7 days (p > 0.05).

**Conclusions:**

The Kinesio™ tape application after 3 and 7 days effectively decreased joint position errors and neck pain intensity in mechanical neck pain participants compared to placebo, while there was no difference between both groups in the NDI.

**Trial registration:**

(CTRI/2011/07/001925). This study was retrospectively registered on the 27th July, 2011.

**Level of evidence:**

**IIB**

## Background

Neck pain is widespread and common in the athletic population, with a lifetime prevalence of 48.3% in specific sports like running, cycling, swimming, football and volleyball [1]. Those who participate in sports that involve maintaining flexed postures for a prolonged time are at higher risk of developing neck pain [2].

In recent years, clinicians have extensively used taping to prevent and treat musculoskeletal injuries in athletes [3]. Kinesio™ tape is a specialized elastic tape that can stretch up to 140% of its resting length and elongate along with the muscle without restricting the joint’s mobility [4, 5]. Kinesio™ tape is water-resistant, thin, and air permeable, as well as adhesive. It places constant shear to the skin because of its adherence properties that mimic those of the skin, and it can be used for 3 to 4 days without removal [6]. One of the proposed mechanisms of Kinesio™ tape is to enhance proprioceptive sensibility after application, as it supports weak muscles, improves muscle functioning, decreases pain, repositions subluxated joints, and facilitates blood and lymph circulation [6–8]. These effects allow affected fascia and muscle to return to normal functioning by decreasing abnormal muscle tension and improving joint function [9].

Kinesio™ tape application is a popular method for rehabilitating athletes with mechanical neck pain. There is a need to collect empirical evidence on how this tape can enhance cervical proprioception. Indeed, to date there is limited evidence on the proprioceptive effect of Kinesio™ tape in athletes with neck pain, thereby formulating a compelling reason to conduct this study. The purpose of this study is to compare the effects of Kinesio™ tape application versus placebo application on cervical proprioception in athletes with mechanical neck pain.

## Methods

### Study design

This study was a randomized, placebo-controlled trial. An independent observer divided all the athletes with mechanical neck pain who met the inclusion criteria into the Kinesio™ tape or placebo groups using a randomization sequence and allocation process. The observer completed this task by using a computer program that was concealed from the primary investigator.

### Participants

Sixty-six multi-sport athletes with mechanical neck pain were referred to the physical therapy clinic by their orthopaedician or general physician. Mechanical neck pain is defined as pain in the cervical and shoulder regions, typically aggravated by neck movements, sustained neck postures, or palpation of the neck muscles [10]. The current study was conducted in the medical rehabilitation department of King Khalid University’s physical therapy clinic, Saudi Arabia, and the data were collected between October 2019 and March 2020. The study only included participants who had been diagnosed with mechanical neck pain, aged above 18 years, and who were willing to participate. The study excluded any participants with a history of whiplash or cervical surgery, diagnosis of fibromyalgia, cervical myelopathy, or any tape allergies, or those who had previous Kinesio™ tape applications to the cervical region. All the participants signed the informed consent document before participation following the Helsinki Declaration. The King Khalid University Ethics and Research Committee board (ECM #2019–61) approved the study. The study trial was registered with ctri.icmr.org.in. Number: CTRI/2011/07/001925.

### Sample size calculation

The study used G*power 3.1 software (Universities, Dusseldorf, Germany) to estimate the sample size for comparing two means: 2-Sample and 2-Sided Equality [11]. We calculated the sample size using the pilot study data for the primary outcome measure, i.e. proprioceptive joint position error (Group A mean = 4.7, Group B mean = 3.7, SD = 1.34), with a power of 0.80 and the alpha value set at 0.05. We estimated the sample size to be 29 participants per group; however, after accounting for dropout rates, we increased the size to 33 participants in each group.

### Outcome measures

All testing took place in a quiet room and lasted for approximately 1 h, all in a single session. The study gave all the participants a trial session before the actual testing to allow them to familiarize themselves with the study protocol and instrumentation. We assessed cervical proprioception with a CROM (cervical range of motion device) device, pain intensity with a VAS (visual analog scale), and functional neck disability with an NDI (neck disability index).

### Cervical proprioception

We estimated cervical proprioception as cervical joint position errors in degrees, adapting the joint position errors testing protocol from Alahmari al.’s study [12]. Moreover, we estimated joint position errors following the subject’s ability to actively reposition their head to a target position that the examiner previously demonstrated. After explaining the testing procedure, the examiner blindfolded the subject with a travel eye mask while the subject sat upright in a chair with their feet flat on the floor and their back straight against the backrest, facing straight ahead. The examiner also used a webbing strap to minimize shoulder and trunk movement during testing. The CROM device was placed on top of the subject’s head and attached posteriorly using a Velcro strap. Each time, the examiner also placed the magnetic yoke squarely over the subject’s shoulders, and the examiner calibrated the CROM device to a neutral position.

For joint position error testing, the examiner guided the subject’s head slowly to the predetermined target position, 50% of the maximum range of motion. Following that, the head remained in the target position for 3 s to allow the subject to memorize the target position. After that, the head was guided back to the neutral position. The examiner then asked the subject to reposition their head independently in the target position. When the subject reached what they believed to be the target position, the examiner measured the subject’s relocation accuracy (joint position errors) in degrees. The speed of active neck motion was kept slow, considering that higher speeds have been associated with significant differences in vestibular function in accordance with age [13, 14]. The examiner measured joint position errors in sagittal and transverse planes (flexion, extension, left rotation, and right rotation). Simultaneously, they randomized the order of testing joint position errors in 4 directions using a simple chit method. Absolute errors, defined as the unsigned difference between the actual angle and the target angle, were recorded as a measurement of cervical joint position errors. The examiner had the participants perform three trials in each direction of movement, and we used the mean of these trials (mean error) for analysis. The baseline proprioception measurements were conducted without tape, while post 3 and 7 days assessments were performed with tape on the subject’s neck.

### Vas

We assessed neck pain intensity using a 10 cm VAS, a horizontal line where 0 indicates no pain, and 10 indicates the worst possible pain that the subject can experience. This scale is a reliable and valid tool to assess pain intensity in a clinical setting [15].

### NDI

In this study, we assessed functional disability due to neck pain using an NDI with well-established reliability and validity [16]. The NDI is a 10-item questionnaire with six possible responses for each item. It is scored from 0 to 50, with a higher score indicating a more significant disability [16]. We converted the NDI scores into percentages by multiplying the total score by 2.

All the outcome measures were recorded at three different times: at the baseline (pre-intervention), after three days, and seven days following the interventions. An independent observer who had no role in the randomization procedure or intervention recorded all the outcome measures.

### Interventions

**Fig. 1:**
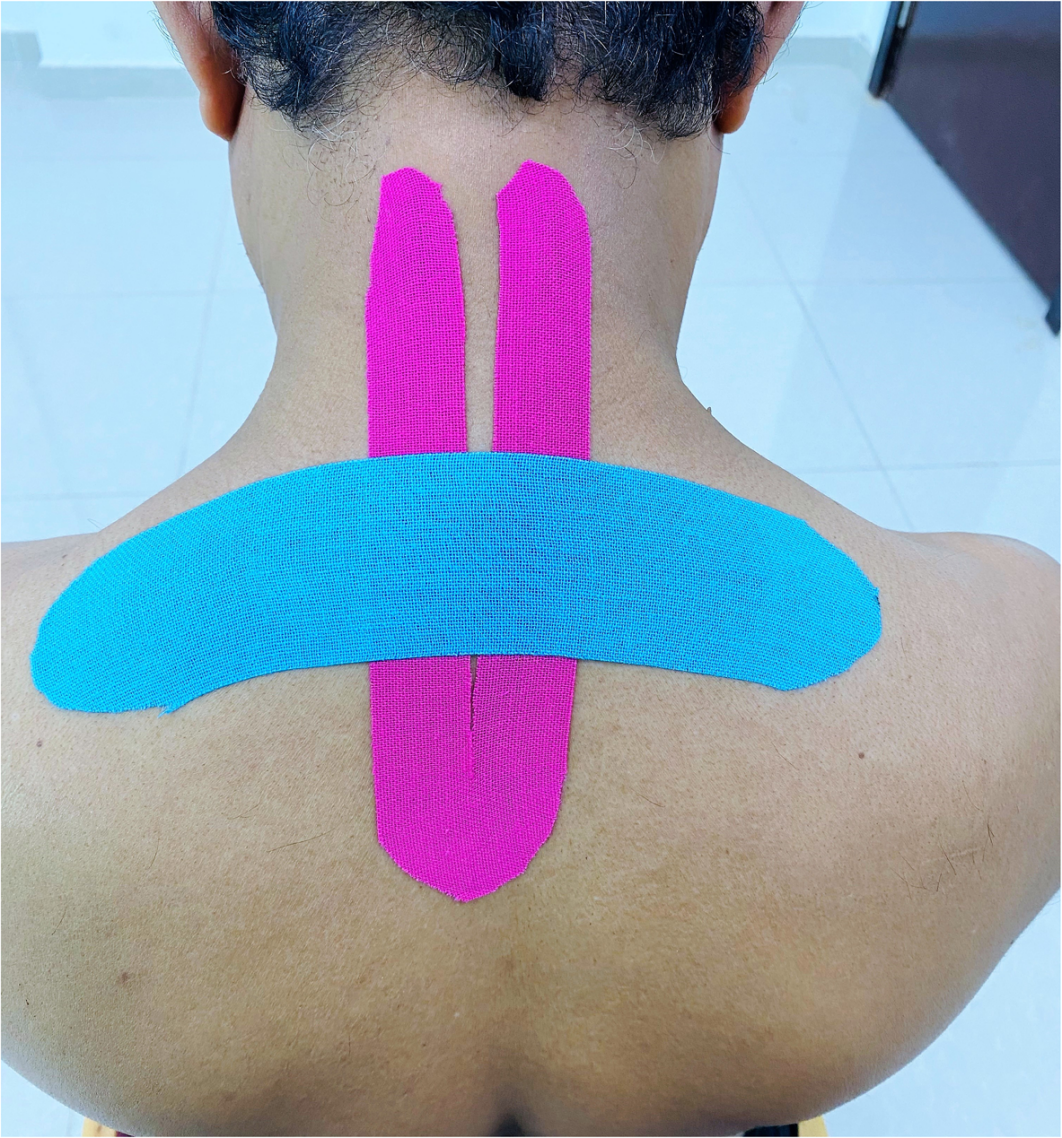
Application of Kinesio tape to Kinesio™ tape group

The Kinesio™ tape (Kinesio Holding Corporation, Albuquerque, NM) used in this study had a thickness of 0.5 mm and a width of 5 cm. It was adhesive, porous, non-allergenic, and waterproof. For the tape application in the Kinesio™ tape group, the subject’s neck was thoroughly cleaned with alcohol and gauze pads before application. The Kinesio™ tape had “Y” and “I” strips applied in 2 layers, according to the GonzáLez-Iglesias et al.’s study guidelines (Fig. 1) [17]. The first layer was a Y-strip: The practitioner places the base, which was un-split, directly over the spine in the mid-thoracic region and stuck without tension. Examiner asked the participants to sit in an upright position with their necks flexed and their chins touching their chests. Following this, the split ends (Y-strip) of the tape were stretched by 15 to 25% of the tape’s resting length and they were pasted up and over either ridge of the spine, covering the cervical musculature. The first strip of Kinesio™ tape extended from T1–T2 of the thoracic region to C1–C2 of the cervical region. The second layer was an overlaying I-strip placed perpendicularly to the Y-strip, covering the maximum posterior cervical musculature at maximum tension to the mid-cervical region (C3–C6). The Kinesio™ tape was stretched from both ends, and the middle portion of the tape was stuck first, after which tension was released to apply the ends without stretching [17, 18]. The placebo group received Kinesio™ tape application with the Y- and I-strips, resembling the real application though with no tension placed on the cervical muscles. Moreover, the practitioner placed the cervical spine of the placebo participants neutrally while applying the tape. The Kinesio™ tape was applied to both groups at the beginning of the testing day and reapplied every two days over the course of a week.

### Statistical analysis

We conducted all statistical analyses of this study using the Statistical Package for Social Sciences Software (SPSS) Version 22.0 (SPSS Inc., Chicago, Illinois, USA). The Shapiro–Wilk test indicated a normal distribution of all study variables. We analyzed demographic data using descriptive statistics, including frequency, percentage, mean, and standard deviation. An independent t-test examined the differences in demographic data between the Kinesio™ tape and placebo groups. A mixed-methods multivariate analysis of variance was used to analyze the differences between the two groups (Kinesio™ tape and placebo) over the 3-time points (baseline and after three days and seven days). In addition, we calculated the effect size as a partial eta squared (ηp^2^), and we set all statistical tests’ significance levels at *P* ≤ 0.05.

## Results

**Fig. 2:**
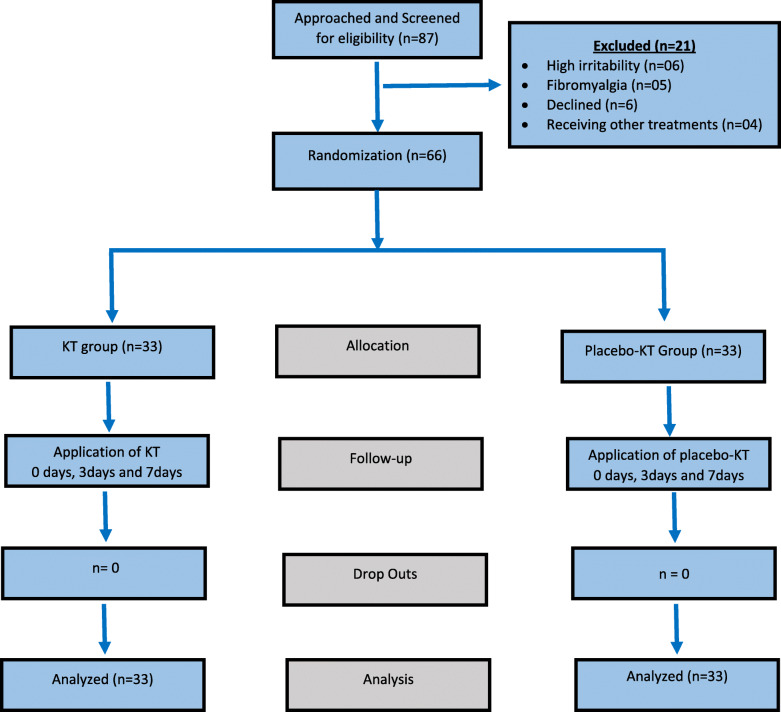
Flow diagram of patients through the trial

Figure 2 presents the flow diagram of participants throughout the trial. Of the 87 participants enrolled in the study, 66 participants met the inclusion criteria, while 21 participants were excluded. 6 participants had radiculopathy symptoms with high irritability; 5 participants had diagnoses of fibromyalgia; 6 participants declined to participate, and four participants were excluded for receiving other treatments. There were no dropouts in this study, and all the participants attended all the sessions. Furthermore, we received no reports of adverse or harmful effects during the study period. Table 1 summarizes the baseline characteristics of the study population. There were no statistically significant baseline differences for age, body mass index, pain intensity, NDI, and proprioception error scores between the Kinesio™ tape and placebo groups (*p* > 0.05). Table 2 exhibits the proprioceptive joint position errors mean and SD values for both groups over the three-time periods (baseline and after 3 and 7 days).

**Table 1:** Demographic characteristics of subjects

Variables	KT group (*n* = 33) Mean ± SD	Placebo-KT (*n* = 33) Mean ± SD	*p*-value
Age (yrs.)	22.73 ± 6.70	23.15 ± 6.64	0.797
BMI (kg/m^2^)	22.73 ± 1.66	23.28 ± 1.86	0.208
Pain Intensity (0 to 10)	05.47 ± 1.44	04.98 ± 1.42	0.167
NDI Score (%)	31.21 ± 8.43	31.27 ± 8.70	0.977
JPE (°)			
Flexion	5.6 ± 1.4	5.1 ± 1.2	0.130
Extension	6.6 ± 1.6	6.3 ± 1.7	0.508
LT Rotation	5.3 ± 1.4	5.1 ± 1.3	0.424
RT Rotation	5.0 ± 1.3	4.7 ± 1.1	0.289

KT Kinesio™ tape, *SD* standard deviation, *BMI* body mass index, *NDI* neck disability index, *JPE* joint position error, *LT* left, *RT* right

**Table 2:** Results of comparison of the outcome measures in both groups and between groups

	KT Group (***n*** = 33)	Placebo Group (***n*** = 33)	MD (95% CI) (lower limit, upper limit)	Cohen’s d	***p***-value
Flexion JPE (^0^)				0.77	
Baseline	5.6 ± 1.4	5.1 ± 1.2	0.5 (− 0.1, 1.1)		0.130
3 days	4.3 ± 1.5	5.0 ± 1.5	0.7 (−1.5, − 0.1)		0.046
7 days	2.6 ± 1.2	4.7 ± 1.4	2.1 (1.4, 2.7)		< 0.001
p-value	< 0.001	0.001			
Extension JPE (^0^)				0.85	
Baseline	6.6 ± 1.6	6.33 ± 1.70	0.2 (−0.5, 1.0)		0.508
3 days	5.5 ± 1.5	6.52 ± 1.50	1.0 (0.2, 1.7)		0.010
7 days	3.5 ± 1.6	6.03 ± 1.94	2.4 (1.7, 3.3)		< 0.001
p-value	< 0.001	0.003			
LT Rotation (^0^)				0.69	
Baseline	5.3 ± 1.4	5.1 ± 1.3	0.2 (−0.4, 0.9)		0.424
3 days	4.3 ± 1.3	4.9 ± 1.5	0.6 (− 0.0, 1.3)		0.064
7 days	3.3 ± 1.0	4.8 ± 1.5	1.4 (0.8, 2.1)		< 0.001
p-value	< 0.001	0.057			
RT Rotation (^0^)				0.59	
Baseline	5.0 ± 1.3	4.7 ± 1.1	0.3 (−0.2, 0.9)		0.289
3 days	4.0 ± 1.2	4.7 ± 1.3	0.6 (0.1, 1.3)		0.034
7 days	2.9 ± 1.0	4.9 ± 1.2	1.9 (1.3, 2.4)		< 0.001
p-value	< 0.001	0.008			
Pain intensity (VAS)				0.57	
Baseline	5.46 ± 1.44	4.97 ± 1.42	0.49 (−0.21, 1.19)		0.167
3 days	4.61 ± 1.40	4.89 ± 1.49	0.26 (− 0.44, 0.98)		0.451
7 days	3.24 ± 0.91	4.64 ± 1.39	1.40 (0.82, 1.98)		< 0.001
p-value	< 0.001	0.102			
Functional Disability (NDI)				0.03	
Baseline	32.18 ± 9.50	31.45 ± 8.64	0.72 (−3.74, 5.19)		0.746
3 days	31.90 ± 9.46	31.21 ± 8.63	0.69 (−3.75, 5.15)		0.756
7 days	31.21 ± 8.43	31.27 ± 8.69	0.06 (−4.15, 4.27)		0.977
p-value	0.009	0.045			

KT Kinesio™ tape, *MD* Mean difference, *JPE* Joint position error, *LT* left, *RT* Right, *VAS* visual analog scale, *NDI* neck disability index

**Fig. 3:**
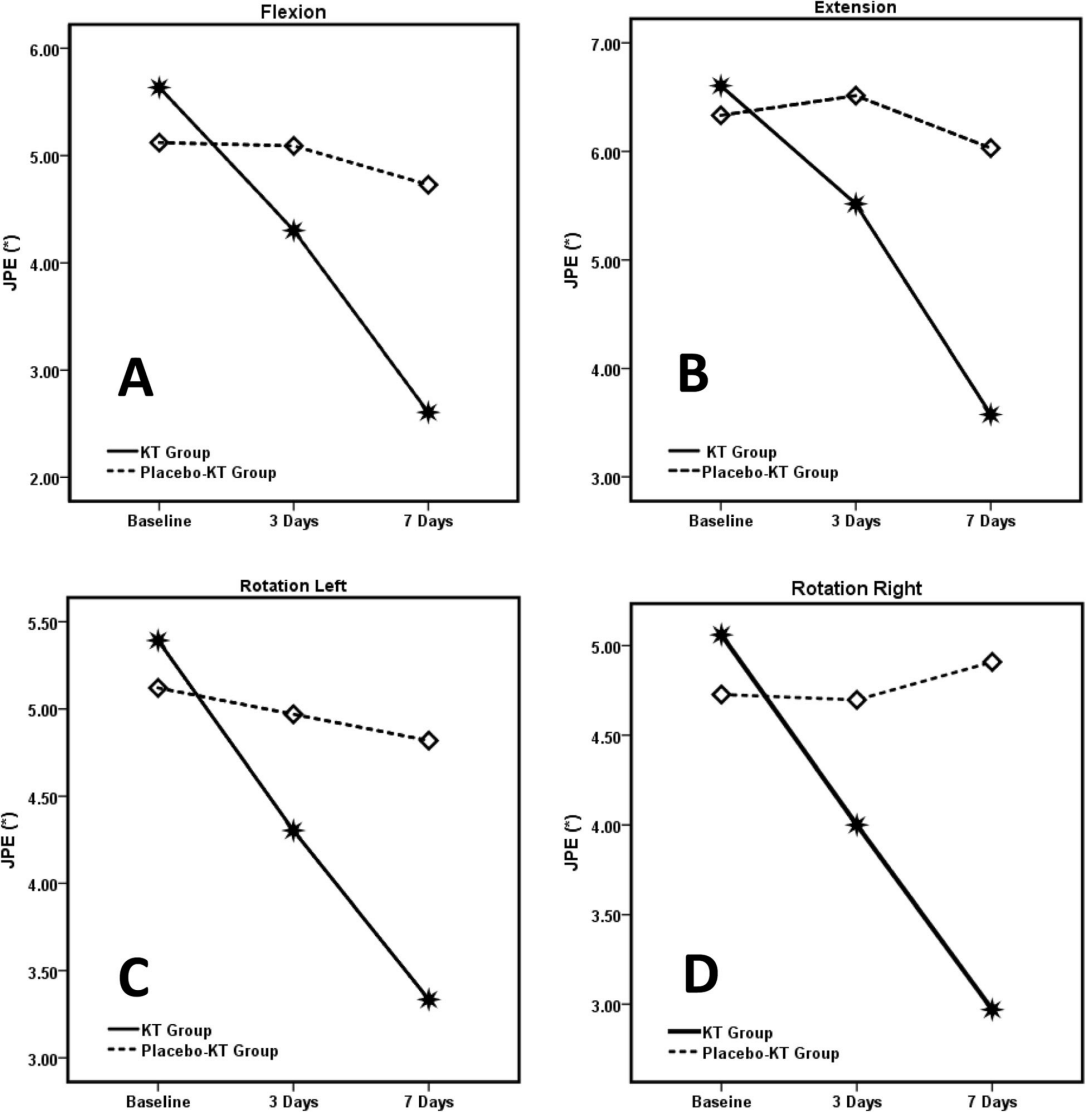
Comparisons of JPE scores between groups and time points

For joint position errors, the results indicated statistically significant group-by-time interaction for flexion (F = 4.15, *p* = 0.046), extension (F = 7.112, *p* = 0.010) and right rotation (F = 4.70, *p* = 0.034) after 3 days of tape application. They indicated significant interaction after 7 days of applying flexion (F = 40.81, *p* < 0.001), extension (F = 31.336, p < 0.001), left rotation (F = 20.743, *p* < 0.001) and right rotation (F = 49.83, p < 0.001). We identified no significant interaction for left rotation (F = 3.55, *p* = 0.064) after applying the tape for 3 days. The Kinesio™ tape group exhibited significant improvement in terms of decreased joint position errors in flexion, extension, left rotation and right rotation by the end of 7 days noticeably more than the placebo group (Table 2 and Fig. 3). The Kinesio™ tape group showed a large (Cohen’s d: JPE in extension: 0.85; flexion: 0.77) to medium-sized (left rotation: 0.69; right rotation: 0.59) improvement regarding joint position errors relative to the placebo group.

Regarding neck pain intensity (VAS), there was statistically significant group-by-time interaction for VAS (F = 23.33, p < 0.001) by 7 days of tape application, and no significant interaction after 3 days of tape application (F = 0.574, *P* = 0.567). The participants in the Kinesio™ tape group experienced a more significant decrease in pain intensity after 7 days of tape application, with a similar improvement in both groups after just 3 days (Table 2). Observed standardized mean differences between the Kinesio™ tape and placebo groups in the change from baseline to follow-up for VAS was medium (Cohen’s d = 0.55).

Concerning functional neck disability, we observe no statistically significant group-by-time interaction for NDI after 3 days (F = 0.09, *p* = 0.756) and 7 days (F = 0.01, *p* = 0.977) of applying the tape. Patients in both groups exhibited similar improvements after 3 days and 7 days of tape application (Table 2). The Kinesio™ tape group showed a small improvement (Cohen’s d = 0.03) in their NDI scores relative to the placebo group.

## Discussion

The current study shows, through its results, that athletes with mechanical neck pain in both groups experienced statistically significant improvements in cervical proprioception after 7 days of applying tape. However, the Kinesio™ tape group exhibited statistically significant improvements regarding decreased joint position errors and pain levels following 3 days and 7 days of applying the tape compared to the placebo group.

The current study’s results follow previous authors’ work who reported proprioception improvement following Kinesio™ tape application [19–22]. Cho et al. showed that proprioceptive sense had enhanced and decreased pain after Kinesio™ tape was applied in a posture-setting exercise on forward head posture for weeks [19]. The minimal clinically important differences (MCID) for proprioception change are 1.5 degrees from the baseline to post-treatment [23, 24]. MCID is the magnitude of change that one must detect before the change surpasses the measurement error [25]. This study showed a change of > 2 degrees from the baseline to 7 days of post- Kinesio™ tape application in all the movement directions we tested.

Several authors have demonstrated improvement in pain following the Kinesio™ tape application [17, 26–30]. GonzáLez–Iglesias et al. applied Kinesio™ tape to patients with acute whiplash who exhibited statistically significant improvements in pain and range of motion immediately following Kinesio™ tape application after a 24-h follow-up [17].

In this study, Kinesio™ tape group demonstrated a change of > 20% from the baseline to 7 days post-intervention compared to the placebo group. This figure surpasses the MCID for pain [31]. Various authors also reported that the Kinesio™ tape application showed no statistically significant benefits. Indeed, the overall effect was possibly too small to be clinically worthwhile [27, 32, 33].

In the current study, the placebo group (without tension) showed improvements in terms of decreased joint position errors (flexion, extension, left rotation). Additionally, we assessed their decrease in pain from the baseline to the 7th-day follow-up. These improvements are perhaps that placebo tape also may have produced mechanical effects that could decrease pain. Considering that the application of Kinesio™ tape was improper, even though it was applied to the cervical muscles, it could have provided sensory feedback during neck movements, thereby decreasing mechanical irritation of soft tissues [34–36]. There may be another explanation: a strong relationship between neck pain intensity and cervical proprioception in participants with neck pain [37], considering that increased pain intensity impairs cervical proprioception and vice versa. As we evidenced, there was a decrease in pain intensity at the 7th-day follow-up. This means that the decreased pain might have positively reduced the magnitude of proprioceptive errors.

Between the group’s comparisons showed a significant baseline to post interventions improvement in VAS score but not in NDI scores. Pain and disability are interrelated, but the relationship between pain and disability in this study is not straightforward. Fejer et al. [38] stated pain and disability were not correlated when the neck pain symptoms were not severe. In this study, participants had mild to moderate pain intensity scores, which may be why NDI scores not showing improvements from baseline to post-intervention. The short-term follow-up could have also been why the NDI’s responsiveness was lower than that of VAS.

For measuring cervical JPE, this study adopted the active head repositioning to the target method, which several authors previously used in clinical settings and was found to be a reliable method [12, 39]. The number of testing trials or movement repetitions in each direction was limited to three to minimize the effect of fatigue of cervical muscles on JPE. Different authors recommended a greater number of trials in each testing direction to improve the reliability of position sense measurement [40], but increasing the number of repetitions can lead to increased pain and fatigability, which may alter the test results of JPEs in participants with neck pain.

### Limitations

This study contains a few limitations. We recruited participants using convenience sampling from one clinic; therefore, these results cannot be generalized to the entire population because they are not representative samples. This study investigated the short-term effects of Kinesio™ tape on cervical proprioception, so we need further studies to observe the long-term impact of Kinesio™ tape.

## Conclusion

Kinesio™ tape application significantly decreased joint position errors and VAS scores at the 3rd-day and 7th-day follow-ups compared to the placebo group. However, we found no differences between the two groups when it came to improving the NDI score after the 3rd-day and 7th-day of tape application. Kinesio™ tape is an adjunct modality that one could use in combination with exercises in treating athletes with mechanical neck pain for better outcomes.

## Data Availability

All data are available at the medical rehabilitation sciences on application to the corresponding author Ravi Shankar Reddy (rshankar@kku.edu.sa).

## Abbreviations

CROM device: Cervical range of motion device
VAS: Visual analog scale
NDI: Neck disability index
